# Burden of Anemia among Human Immunodeficiency Virus-Positive Adults on Highly Active Antiretroviral Therapy at Hawassa University Compressive Specialized Hospital, Hawassa, Ethiopia

**DOI:** 10.1155/2023/2170447

**Published:** 2023-10-14

**Authors:** Sisay Tesfaye, Melaku Hirigo, Dawit Jember, Mekdes Shifeta, Worku Ketema

**Affiliations:** ^1^Hawassa University, College of Medicine and Health Science, Faculty of Medicine, Department of Internal Medicine, Hawassa, Ethiopia; ^2^Hawassa University, College of Medicine and Health Science, School of Public Health, Hawassa, Ethiopia; ^3^Hawassa University, College of Medicine and Health Science, Faculty of Medicine, Department of Pediatrics and Child Health, Hawassa, Ethiopia

## Abstract

**Background:**

Anemia is the most common hematologic abnormality associated with human immunodeficiency virus (HIV)-infected patients and affects 60% to 80% of patients in late-stage disease. It has a considerable impact on the progression of HIV to advanced stages. This study aimed at assessing the burden of anemia in adult HIV-infected patients who are on highly active antiretroviral therapy (HAART) and have follow-up at Hawassa University Comprehensive Specialized Hospital (HUCSH) Antiretroviral therapy (ART) clinic.

**Methods:**

A hospital-based retrospective study was conducted among HIV-positive adults on HAART at Hawassa University Compressive Specialized Hospital. The systematic sampling method was used to choose a total of 244 study participants. Data on demographic characteristics, related factors of anemia, latest hemoglobin, CD4, and ART regimens were collected using a structured data abstraction format. The data were cleaned and analyzed using SPSS version 21.0 after being manually checked for completeness. Multivariable logistic regression was carried out to detect elements associated with anemia. A *P* value of <0.05 was used as a cutoff point to announce statistical significance.

**Results:**

The records of 244 patients were examined in total. Anemia was present in 29.9% (95% CI 23.8–35.2) among adult HIV patients. Female sex (AOR: 2.576, 95% (CI: 1.295–5.127)), having tuberculosis (TB) (AOR: 4.873, 95% (CI: 1.534–15.484)), taking a zidovudine (ZDV)-containing ART regimen (AOR: 5.216, 95% (CI: 1.239–21.962)), having clinical WHO stage IV and III diseases (AOR: 3.077, 95% CI (1.244–7.612)), having body mass index (BMI) <18.5 kg/m^2^ (AOR: 2.391, 95% (CI: 1.138–5.023)), and taking cotrimoxazole prophylaxis (AOR: 3.860 95% (CI: 1.097–13.576)) were substantially linked to the development of anemia among adult HIV patients. *Conclusion and Recommendation*. This study showed that anemia is still a problem among HIV patients on HAART. The burden of anemia was found to be high among patients with advanced WHO clinical stages, having a BMI less than 18.5 kg/m^2^, TB/HIV coinfection, being on AZT-based ART regimens, and taking cotrimoxazole preventive therapy (CPT). Consequently, it is suggested that early preventative interventions, such as serial hemoglobin follow-up, iron supplementation, and education about dietary consumption, be undertaken targeting the aforementioned groups. In addition, the preferred first-line ART regimen as per the latest national and WHO guidelines is recommended, especially for the above groups.

## 1. Introduction

The human immunodeficiency virus (HIV) is a virus that affects the human immune system and puts the infected person at risk of opportunistic infections. Acquired immunodeficiency syndrome (AIDS) is the late stage of the disease. The current 2021 UNAIDS Ethiopia Spectrum national PLHIV estimate is 616,105 [1].

Hematological abnormalities manifested by pancytopenia are among the most common manifestations of HIV/AIDS, with anemia being the leading abnormality [2–4]. Anemia is a state characterized by a decreased quantity of red blood cells that is not enough to meet the body's physiological requirements [5]. However, the distribution of anemia in HIV patients varies significantly, ranging from 1.3% to 95% [6]. There are a lot of factors, including stage of HIV, age, and sex, which are said to account for the variations in the distribution of anemia in HIV-infected individuals [6]. Direct effects of the virus on bone marrow, medication related, poor intake result in nutritional deficiency, and anemia of chronic illnesses are common reasons [7, 8]. Normocytic normochromic anemia is the most encountered morphologic type of anemia in HIV, followed by microcytic hypochromic and macrocytic anemia [9, 10]. Anemia of chronic disease is the most common cause of normocytic normochromic and microcytic hypochromic anemia in HIV-infected individuals, and iron deficiency anemia is a relatively infrequent cause of microcytic hypochromic anemia [9]. The natural course of HIV disease influenced by anemia increases the rate of disease progression to advanced stage and increase mortality [11]. Severe anemia is associated with a faster rate of HIV disease progression. They have also demonstrated that anemia is an important indicator of death among HIV patients. Untreated anemia can exacerbate poverty in areas with a high HIV incidence and cause a variety of systemic consequences such as fatigue, exhaustion, a higher risk of HIV dementia, poor quality of life, and more [12]. On the other hand, survival time in HIV-infected persons may be improved after recovery from anemia [13]. Globally, anemia is a major health problem that can cause poor quality of life, morbidity, and death and affects the socioeconomic development of a nation. It has a greater impact on developing countries than on developed countries [14].

Despite the fact that low level of red blood cell mass is a major cause of morbidity and mortality in patients with HIV/AIDS, data on anemia and associated risk factors among RVI patients are generally insufficient from low-resource countries such as Africa, including Ethiopia. It is still a common problem among patients taking ART, so early identification and interventions to correct anemia may lead to improved health and survival potential of HIV-infected people. Identification and correction of associated factors will help to prevent the development of anemia, which in turn will improve the quality of life and survival of HIV/AIDS patients.

We conducted this study to determine the burden of anemia among adult HIV patients receiving ART at the ART clinic at Hawassa University Comprehensive Specialized Hospital because, to the best of our knowledge, there are no published data on this topic in the study area.

## 2. Methods

### 2.1. Study Setting and Period

The study was carried out at Hawassa University Comprehensive Specialized Hospital, Hawassa City, Sidama Region, Ethiopia. Hawassa University Comprehensive Specialized Hospital provides HIV/AIDS interventions, including free diagnosis, treatment, and follow-up. ART clinic is a major unit under the department of internal medicine. It was established in the hospital in 1997, E.C., and since then the hospital has provided ART services to 7965 clients. Among this group, there are 2,802 currently active adult clients on ART. The center diagnoses new cases and follows those on therapy. Structured HIV/AIDS data are available at this specialized hospital.

This study was carried out from August to December 2021 to search for the burden of anemia among people with HIV/AIDS who are on ART at HUCSH.

### 2.2. Study Design

A facility-based retrospective cross-sectional study was conducted among HIV-positive adults at the ART follow-up clinic of HUCSH.

### 2.3. Source Population

Source population includes all HIV-positive patients above the age of 18 years having follow-up at ART clinic at Hawassa University Comprehensive Specialized Hospital.

### 2.4. Study Population

Study population includes all HIV positive patients above the age of 18 years on ART who had follow-up at ART clinic during the study period at Hawassa University Comprehensive Specialized Hospital.

### 2.5. Inclusion and Exclusion Criteria

#### 2.5.1. Inclusion Criteria

The inclusion criteria were HIV-positive patients aged 18 years or older on ART, who have had regular follow-up and latest laboratory investigation (complete blood count (CBC) within 3 months and CD4 count within 6–12 months).

#### 2.5.2. Exclusion Criteria

Patients transferred from other setups to the HUCSH ART clinic on their first visit, pregnant women and women in the postpartum period, and patients with incomplete information for hemoglobin (Hgb) status on the chart during follow-up were excluded from the study.

### 2.6. Sample Size Determination and Sampling Procedure

#### 2.6.1. Sample Size Determination

A single population proportion formula was used to estimate the sample size with the following assumptions: prevalence of anemia after initiation of ART is 80.5% [15], the margin of error is 5%, and the confidence interval is 95% [15]. Therefore, the sample size required for this study was 244 patients.

#### 2.6.2. Sampling Method

Among those adults attending the ART clinic at HUCSH during the study period, a total of 244 HIV-positive adults taking ART and on follow-up was selected by systematic sampling technique.

### 2.7. Data Collection Methods and Tools

The data were collected using a chart review by the nurses and physicians working in the ART clinic. A total of three health professionals, one ART nurse, one general practitioner as data collector, and one resident as supervisor from another unit, participated in the data collection process of this study. The information collected includes sociodemographics, clinical characteristics, and the immunohematology profile of patients. Data on the sociodemographic and clinical characteristics of the study participants were collected through a review of medical records. Weight and height measurements were taken from patients' charts, and BMI was calculated by dividing weight in kg by height in m^2^. The BMI cutoff value according to WHO classification (in kg/m^2^) was <18.5 (underweight), 18.5–24.99 (normal), 25–29.99 (overweight), and ≥30 (obese) [16].

### 2.8. Study Variables

#### 2.8.1. Dependent Variable

Dependent variable is anemia.

#### 2.8.2. Independent Variables

1. Sociodemographic factors: age, sex, level of education, place of residence, occupation, marital status, and dietary intake
2. WHO stage of HIV
3. CD4+ T-cell count was taken from patient records and classified as <200 cells/*µ*L, 200–500 cells/*µ*L, and ≥500 cells/*µ*L based on CDC
4. Nutritional status (BMI)
5. ART regimen

### 2.9. Operational Definitions

1. Anemia is defined as hemoglobin (Hgb) concentration less than 13 g/dl for adult males and less than 12 g/dl for adult females [17, 18]
2. Mild anemia is defined as Hbg 11–11.9 g/dl for women and 11–12.9 g/dl for men
3. It is moderate (Hbg 8–10.9 g/dl) and severe (Hgb < 8 g/dl) for both sexes [14, 18]
4. Microcytosis is defined as MCV <80 fL and macrocytosis is MCV >100 fl [18]
5. Anemia prevalence cutoff values for public health significance include the following: <5%, no public health problem; 5–19%, mild public health problem; 20–39%, moderate public health problem; and ≥40%, severe public health problem [19]

### 2.10. Data Management and Analysis

The data were first checked manually for completeness and then entered and analyzed using SPSS version 21. Univariate analysis was used to summarize descriptive measures. Bivariate logistic regression analysis was used to identify candidate variables for multivariable logistic regression analysis. The multivariable analysis was conducted to control (adjust) for possible confounding variables. During the analyses, a *P* value <0.05 with 95% confidence interval (CI) for AOR (adjusted odds ratio) was used in judging the significance of the associations. Results were presented in text and tables.

### 2.11. Data Quality Control

To ensure the quality of the data, prior to data collection, training of the data collectors was carried out for one day by the principal investigator on the objective, relevance of the study, and confidentiality of information. The abstraction format was prepared in English for better understanding. The questionnaire was pretested with a 5% of sample size to learn about the appropriateness of the questions, which was conducted at the Adare General Hospital ART clinic, another public hospital in Hawassa City.

### 2.12. Ethical Considerations

Ethical clearance was obtained from the institutional review board of HUCSH. Then, permission was obtained from hospital management. After permission was obtained, data were collected from the ART clinic.

## 3. Result

### 3.1. Sociodemographic Characteristics

Out of two hundred forty-four patients reviewed, one hundred thirty-five (55.3%) of the study participants were females, of whom 21.7% were anemic, and the remaining, one hundred nine (44.7%) were males. Most participants, one hundred twenty-eight (52.4%), were in the age of 18–25 years.

Regarding the marital status of the participants, one hundred fifty-five (63.5%) were married, sixty-four (26.2%) were single, fifteen (6.1%) were divorced, and ten (4.1%) were widowed. The majority of participants, one hundred sixty-six (68%), were urban residents, and the rest seventy-eight (32%) were from rural areas. When it comes to the participants' religion, 117 patients (47.9%) were identified as orthodox, whereas eight (36%), twenty-three (9.5%), and sixteen (6.6%) were identified as protestants, Muslims, and others, respectively. Regarding their occupation, ninety-two (37.7%) were private workers and twenty-seven (11.1%), seventy-two (29.5%), and fifty-three (21.8%) were daily laborers, farmers, and government employees, respectively. In terms of educational attainment, thirty-three (13.5%), sixty-two (25.4%), seventy (28.7%), thirty-nine (16%), and forty (16.45) were illiterate, could read and write, and attended primary level, secondary level, and higher level education consecutively (Table 1).

### 3.2. Baseline Clinical Characteristics

The large proportion of the study participants, two hundred ten (86.1%), was in WHO clinical stages of I and II, and the rest thirty-four (13.9%) were in WHO clinical stages III and IV. Two hundred thirty (94.3%) of the participants were taking non-AZT-containing regimen, and the remaining fourteen (5.7%) patients were on AZT-containing regimens.

About one hundred twenty (49.2%) of the study participants had a CD4 count of ≥500 cells/*µ*L, and eighty-two (33.6%) and forty-two (17.2%) of the participants had a CD4 count between 200 and 500 cells/*µ*L and a CD4 count below 200 cells/*µ*L, respectively. Regarding tuberculosis, twenty-two (9.1%) of the study participants had a history of tuberculosis infection. About one hundred ninety (77.9%) of participants were not on cotrimoxazole and the remaining fifty-four (22.1%) were taking cotrimoxazole prophylactic therapy.

In terms of HIV duration from diagnosis, one hundred participants (41%) had HIV for 1–5 years, while fifty-seven (23.4%), forty-one (16.8%), and forty-six (18.8%) had HIV for 6–10 years, more than ten years, and 6 months to one year consecutively. Most patients, one hundred (41%) took ART for one to five years. The majority of patients, one hundred eighty-three (75%), had a body mass index of >18.5 kg/m^2^, and the remaining sixty-one (25%) had ≤18.5 kg/m^2^ (Table 2).

### 3.3. Magnitude of Anemia

Anemia was detected in 29.9% (95% CI 23.8–35.2) of HIV adult patients receiving ART. Thirty-three (13.5%), thirty-six (14.7%), and four (1.7%) of the patients had mild anemia, moderate anemia, and severe anemia, respectively. Thirty-four of these patients (13.9%) had normocytic anemia, thirty-three (13.5%) had macrocytic anemia, and six (2.5%) had microcytic anemia, according to the morphologic type of their anemia.

### 3.4. Factors Associated with Anemia among Adult HIV-Positive Patients

Bivariate logistic regression took into account a total of 15 variables, and 7 variables were included in the multivariable logistic regression model. Accordingly, females were three times more likely to develop anemia than males (AOR): 2.576, 95% CI (1.295–5.127). Participants with tuberculosis (TB) were five times more likely to develop anemia than those who did not (AOR: 4.873, 95% CI (1.534–15.484)). Those patients on zidovudine (ZDV)-containing ART regimens were five times more likely to develop anemia than those on non-AZT-containing regimens (AOR: 5.216, 95% CI (1.239–21.962)).

Participants with clinical WHO stages III and IV were three times more likely to develop anemia than participants with clinical WHO stages I and II (AOR: 3.077, 95% CI (1.244–7.612)). Participants with a body mass indexes (BMIs) of less than or equal to 18.5 kg/m^2^ were two times more likely to develop anemia than those having BMI of more than 18.5 kg/m^2^ (AOR: 2.395 (1.138–5.023)). Patients who were on cotrimoxazole prophylactic therapy were four times more likely to develop anemia than their counterparts (AOR; 3.860, 95% CI (1.097–13.576)) (Table 3).

## 4. Discussion

The burden of anemia in our study (29.9% (95% CI 23.8–35.2)) is comparable with the findings of a similar study carried out in other areas (31.1% in the study conducted in Kambata, southern Ethiopia [20] and 26.2% in the study conducted in South Africa [21]). However, it is lower than that of studies conducted in other parts of the country: Zewidtu Memorial Hospital (42.9%) [20] and Tikur-Anbessa Specialized Hospital (41.9%) [21]. In addition, it was lower than the results of studies undertaken in other African countries: Ghana (63%) [2] and Tanzania (40.7%) [22]. The burden discovered in the current study, however, was higher than that of investigations carried out at Jimma University Hospital (16.2%) [23, 24] and in Malawi (16.2%) [14]. The disparities in the burden of anemia may be explained by the differences in the study area, study time, and health intervention measurement undertaken. Being a woman was highly related with anemia in the current study, which is consistent with the majority of other studies [4, 24] and reference [25]. Obstetric and gynecological conditions such as menstruation, pregnancy, and lactation may be the cause for this [14].

Another factor in the current study that is strongly linked to the burden of anemia is the use of ART medications that contain AZT. Studies carried out in Tanzania [22] and Kambata, South Ethiopia [25], backed up this conclusion. This is due to the fact that zidovudine suppresses erythroid stem cells, which inhibits the erythropoiesis process (red blood cell production) in the bone marrow and, as a result, lowers the generation of reticulocytes and hemoglobin levels [22].

This study discovered that having a WHO clinical stage III or IV considerably increased the risk of anemia, which is consistent with other studies carried out in Tanzania [22], Kamabata, south Ethiopia [25], and Zewidtu Memorial Hospital [20]. This is brought on by chronic inflammation and high viral replication that characterize the advanced stage of HIV. In other words, the high-viral load at the late stage of HIV causes defective hematopoiesis and altered coagulation processes, which cause the level of hemoglobin to decrease during this time and cause anemia to develop [26].

This study found that having tuberculosis increased the likelihood of anemia. Study reports from Kambata, south Ethiopia [25], and northwest Ethiopia [23] corroborated this conclusion. This is due to the immune system's reaction to tuberculosis, which causes the release of cytokines that impair the body's capacity to use both stored iron and iron obtained from nutrition, as well as the creation and regular function of the hormone erythropoietin. Anemia of chronic disease was the main cause for underlying anemia in HIV-TB coinfected patients [27].

Participants in this study were shown to have a higher risk of anemia if their BMI was under 18.5 kg/m^2^. This result is consistent with study reports from Malawi, Kambata, south Ethiopia, and northwest Ethiopia [14, 23, 25]. A lower BMI indicates under nutrition as a result of insufficient calorie intake, poor nutrient absorption, or poor nutrient utilization, resulting in a deficiency of iron, folate, and vitamin B-12 for erythrocyte production. Furthermore, it increases the risk of infection, which increases the possibility of anemia [28].

This study discovered that cotrimoxazole use is connected with the development of anemia, which is consistent with similar study reports from the Ayder Mekelle specialized hospital [29] and study reports from Cote d'Ivoire [30]. The association between CPT and anemia may be explained by trimethoprim, a mild dihydrofolatereductase inhibitor that can limit folic acid metabolism and, at high doses, produce megaloblastic changes, especially in people who are not taking supplemental folate [31].

## 5. Limitations of the Study

This paper has its own limitations, such as the lack of information on dietary diversities, issues such as menstrual cycles, and workups for other causes of anemia. The social desirability bias, which we tried to minimize during data collection, was another limitation of our study.

## 6. Conclusion and Recommendation

This study showed that anemia is still a problem among HIV patients on HAART. The burden of anemia was found to be high among patients with advanced WHO clinical stages, having a BMI less than 18.5 kg/m^2^, TB/HIV coinfection, being on AZT-based ART regimens, and taking cotrimoxazole preventive therapy (CPT). Consequently, it is suggested that early preventative interventions, such as serial hemoglobin follow-up, iron supplementation, and education about dietary consumption, be undertaken targeting the aforementioned groups. In addition, the preferred first-line ART regimen as per the latest national and WHO guidelines is recommended, especially for the above groups.

## Figures and Tables

**Table 1:** Socioeconomic and demographic characteristics of adult HIV patients on HAART at Hawassa university specialized hospital, 2021 (*n* = 244).

Background characteristics	Not anemic *N* (%)	Anemic *N* (%)	COR (95% CI)
*Sex*			
Male	89 (36.5%)	20 (8.2%)	Ref
Female	82 (33.6%)	53 (21.7%)	2.876 (1.586–5.217)

*Age*			
18–25	85 (34.8%)	43 (17.6%)	0.843 (0.192–3.695)
26–33	36 (14.8%)	14 (5.7%)	0.648 (0.136–3.081)
34–41	23 (9.4%)	10 (4.1%)	0.725 (0.144–3.634)
42–49	22 (9%)	3 (1.2%)	0.227 (0.35–1.477)
50 and more	5 (2%)	3 (1.2%)	Ref

*Residency*			
Urban	118 (48.45)	48 (19.7%)	1.160 (0.648–2.075)
Rural	53 (21.7%)	25 (10.2%)	Ref

*Occupation*			
Daily laborer	21 (8.6%)	6 (2.5%)	Ref
Farming	50 (20.5%)	22 (9%)	1.540 (0.546–4.342)
Private work	63 (25.8%)	29 (11.9%)	1.611 (0.588–4.416)
Gov. employee	37 (15.2%)	16 (6.6%)	1.514 (4.458)

*Religion*			
Protestant	63 (25.8%)	25 (10.2%)	Ref
Orthodox	83 (34%)	34 (13.9%)	1.032 (0.560–1.903)
Muslim	16 (6.6%)	7 (2.9%)	1.102 (0.405–3.002)
Others	9 (3.7%)	7 (2.9%)	1.960 (0.658–5.835)

*Marital status*			
Single	44 (18%)	20 (8.2%)	Ref
Married	108 (44.3%)	47 (19.3%)	0.957 (0.510–1.797)
Divorced	10 (4.1%)	5 (2%)	1.100 (0.332–3.640)
Widowed	9 (3.7%)	1 (0.4%)	0.244 (0.29–2.062)

*Educational status*			
Illiterate	23 (9.4%)	10 (4.1%)	Ref
Can read and write	42 (17.2%)	20 (8.2%)	1.095 (0.439–2.731)
Primary education	48 (19.7%)	22 (9%)	1.054 (0.430–2.587)
Secondary education	28 (11.5%)	11 (4.5%)	0.904 (0.326–2.502)
Higher level education	30 (12.3%)	10 (4.1%)	0.767 (0.273–2.150)

**Table 2:** Baseline clinical characteristics of adult HIV patients on HAART at Hawassa university specialized hospital, 2021 (*n* = 244).

Background characteristics	Not anemic *N* (%)	Anemic *N* (%)	COR (95% CI)
*TB*			
No	165 (67.6%)	57 (23.3%)	Ref
Yes	6 (2.5%)	16 (6.6%)	7.719 (2.882–20.679)

*BMI*			
>18.5	144 (59%)	39 (16%)	Ref
≤18.5	27 (11.1%)	34 (13.9%)	4.650 (2.509–8.616)

*ART regimen*			
Non-AZT-containing	168 (68.9%)	62 (25.4%)	9.935 (2.682–36.801)
AZT-containing	3 (1.2%)	11 (4.5%)	Ref

*Duration of ART*			
6 months–1 year	31 (12.7%)	17 (7%)	Ref
1–5 years	74 (30.3%)	26 (10.7%)	0.641 (0.305–1.345)
6–10 years	37 (15.2%)	18 (7.4%)	0.887 (0.392–2.008)
>10 years	29 (11.9%)	12 (4.9%)	0.755 (0.308–1.848)

*Duration of HIV*			
6 months–1 year	29 (11.9%)	17 (7%)	Ref
1–5 years	75 (30.7%)	25 (10.2%)	0.569 (0.268–1.204)
6–10 years	38 (15.6%)	19 (7.8%)	0.853 (0.378–1.924)
>10 years	29 (11.9%)	12 (4.9%)	0.706 (0.287–1.737)

*WHO stage*			
Stage I &II	154 (63.1%)	56 (23%)	Ref
Stage III & IV	17 (7%)	17 (7%)	2.750 (1.314–5.756)

*Cotrimoxazole*			
No	147 (60.2%)	43 (17.6%)	Ref
Yes	24 (9.8%)	30 (12.3%)	4.273 (2.264–8.066)

*CD4 counts*			
≥500	97 (39.8%)	23 (9.4%)	Ref
200–500	55 (22.5%)	27 (11.1%)	2.070 (1.084–3.954)
<200	19 (7.8%)	23 (9.4%)	5.105 (2.390–10.904)

*Mean cell volume*			
<70	10 (4.1%)	6 (2.5%)	Ref
70–100	84 (34.4%)	34 (13.9%)	0.675 (0.227–2.002)
>100	77 (31.6%)	33 (13.5%)	0.714 (0.240–2.127)

**Table 3:** Factors associated with anemia among adult HIV patients on HAART at Hawassa university comprehensive specialized hospital, Hawassa, Ethiopia, 2021 (*n* = 244).

Characteristics	Anemia status			
	Not anemic *N* (%)	Anemic *N* (%)	COR (95% CI)	AOR (95% CI)
*Sex*				
Male	89 (36.5%)	20 (8.2%)	Ref	Ref
Female	82 (33.6%)	53 (21.7%)	2.876 (1.586–5.217)	2.576 (1.295–5.127)

*ART regimen*				
Non-AZT-containing	168 (68.9%)	62 (25.4%)	9.935 (2.682–36.801)	Ref
AZT-containing	3 (1.2%)	11 (4.5%)	Ref	5.216 (1.239–21.962)

*TB*				
No	165 (67.6%)	57 (23.3%)	Ref	Ref
Yes	6 (2.5%)	16 (6.6%)	7.719 (2.882–20.679)	4.873 (1.534–15.484)

*BMI*				
>18.5	144 (59%)	39 (16%)	Ref	Ref
≤18.5	27 (11.1%)	34 (13.9%)	4.650 (2.509–8.616)	2.391 (1.138–5.023)

*WHO stage*				
Stage I &II	154 (63.1%)	56 (23%)	Ref	Ref
Stage III & IV	17 (7%)	17 (7%)	2.750 (1.314–5.756)	3.077 (1.244–7.612)

*Cotrimoxazole*				
No	147 (60.2%)	43 (17.6%)	Ref	Ref
Yes	24 (9.8%)	30 (12.3%)	4.273 (2.264–8.066)	3.860 (1.097–13.576)

## Data Availability

The data are fully available upon a reasonable request from the correspondent author.
